# Supplementary material for: Living with depression and diabetes: A qualitative study in Bangladesh and Pakistan
Source: PLOS Glob Public Health. 2024 Jan 25;4(1):e0002846. doi: 10.1371/journal.pgph.0002846 (PMC10810509; doi:10.1371/journal.pgph.0002846)
Supplement: Attachment — Submitted filename: Response to Reviewers.pdf [file pgph.0002846.s002.pdf]

Department of Health Sciences
University of York
Heslington YO10 5DD

23rd May 2023

Dear Editor,

I am pleased to re-submit a revised version of the manuscript "Depression and diabetes in Bangladesh and Pakistan: a qualitative study exploring the feasibility and acceptability of integrating Behavioural Activation for depression into diabetes care".

We appreciate the time taken to review the paper and we have carefully considered all the points made and revised the paper accordingly. We believe the paper is a much stronger as a result. Below are the responses to each point raised by the editor and reviewers.

We hope that the paper is now acceptable for publication. We look forward to sharing its finding with the readers of PLOS Global Public Health.

Yours faithfully,

Hannah Jennings, PhD
(on behalf of all the authors)

Editor/Journal requirements

1. Please amend your detailed Financial Disclosure statement. This is published with the article. It must therefore be completed in full sentences and contain the exact wording you wish to be published.
a. State the initials, alongside each funding source, of each author to receive each grant.
b. State what role the funders took in the study. If the funders had no role in your study, please state: "The funders had no role in study design, data collection and analysis, decision to publish, or preparation of the manuscript."
c. If any authors received a salary from any of your funders, please state which authors and which funders. If you did not receive any funding for this study, please simply state: "The authors received no specific funding for this work."

We have amended the financial disclosure section as requested. It now reads:

"This work is part of the DiaDeM programme, funding was received from the National Institute for Health and Care Research (NIHR) Research and Innovation for Global Health Transformation (RIGHT) funding programme, grant number NIHR200806. NS is the PI of DiaDeM and HMJ, SA and DE are named co-investigators. All co-authors (HMJ, SS, AN, SA, NA, FA, GZG, NS and DE) received funding from this award. The funder had no role in the study design, data collection, analysis or writing of the paper."

2. Your manuscript is missing the following sections: Introduction. Please ensure these are present, and in the correct order, and that any references to subheadings in your main text are correct.

We have amended as suggested. There is now a clearly labelled introduction.

3. We have noticed that you have uploaded Supporting Information files, but you have not included a list of legends. Please add a full list of legends for your Supporting Information files after the references list.

A list of legends has been added as requested.

4. In the online submission form, you indicated that "Data may be available upon reasonable request to the Principle Investigator of DiaDeM (NS) and lead for this study (HMJ)". All PLOS journals now require all data underlying the findings described in their manuscript to be freely available to other researchers, either 1. In a public repository, 2. Within the manuscript itself, or 3. Uploaded as supplementary information. This policy applies to all data except where public deposition would breach compliance with the protocol approved by your research ethics board. If your data cannot be made publicly available for ethical or legal reasons (e.g., public availability would compromise patient privacy), please explain your reasons by return email and your exemption request will be escalated to the editor for approval. Your exemption request will be handled independently and will not hold up the peer review process, but will need to be resolved should your manuscript be accepted for publication. One of the Editorial team will then be in touch if there are any issues.

We are unable to make the raw data publicly available due to ethical restrictions. As advised we contacted PLOS Global Health and explained the restrictions. We received a response from the editor stating "Your paper would be considered compliant with PLOS policy if you include in your Data Availability an explanation of the ethical restrictions on your data and a point of contact for the IRB or REB who granted ethical approval to your study." We have now explained the ethical restrictions on making the data available and added a point of contact for the board that granted the ethics approval.

5. The manuscript will require a major re-working to address the fundamental issues raised by Reviewer 1.

We have tried to address all of reviewer 1's concerns – our responses are below.

Reviewer 1

The authors have conducted a qualitative study in Pakistan and Bangladesh to explore experiences of depression and diabetes and to assess the feasibility and acceptability of a behavioural activation for depression in the diabetes treatment. The title as well as the objectives suggest that the main focus of the study is the feasibility and acceptability of BA intervention in this context. However, a large portion of the section is dedicated to describing the experience of depression in diabetes. The way the verbatim quotes have been presented and results handled in this section is also vague and non specific to the lived experiences. The feasibility and acceptability of the behavioural activation have not been explored in detail and have been presented superficially. The feasibility is presented in terms of space availability and resource availability issues. Acceptability has been explored very superficially. Given these limitations, I believe that the study has not achieved the objectives that it proposed.

We thank you for your comments. We have considered the comments in detail - by doing this we have been able to reflect on the paper, clarify what was done, made some changes and strengthened it. We have summarised changes below, responding to each concern.

"The title as well as the objectives suggest that the main focus of the study is the feasibility and acceptability of BA intervention in this context. However, a large portion of the section is dedicated to describing the experience of depression in diabetes."

The paper was reporting on a qualitative study that has informed the adaptation of a BA intervention in Bangladesh and Pakistan, and is part of a larger programme of research (DiaDeM). In order to explore the feasibility of BA and identify how it might be optimised we needed to understand the context (i.e. the experiences of depression in diabetes and care-seeking from provider and patient perspectives). In the introduction we have clarified why it is important to explore context (lines 93-95). The objectives of the research make it clear we are: exploring the lived experiences of people with diabetes and depression (objective 1) and clinical practice and care seeking (objective 2) (context), as well as exploring the feasibility and acceptability of BA (objectives 3).

"The way the verbatim quotes have been presented and results handled in this section is also vague and non specific to the lived experiences."

We have reviewed the results and quotes. The quotes are verbatim representing a mix of participants and were selected carefully to illustrate each part of the results. We needed to limit the number of the quotes due to the length of the paper, and we were careful to keep them non-identifiable. Reviewer 2 has commented on the analysis: "This paper reads extremely well. All sections are well written and the data collection and analysis were well executed."

"The feasibility and acceptability of the behavioural activation have not been explored in detail and have been presented superficially. The feasibility is presented in terms of space availability and resource availability issues. Acceptability has been explored very superficially."

We disagree that feasibility and acceptability of BA were not explored in detail. As described in the paper, the topic guide was thorough covering context, experiences and views on 'talking therapy'. An

explanation of BA was developed and piloted in both Pakistan and Bangladesh in order to ensure it was understood when asked about as part of the interview. BA theory was drawn on and participants were probed about the link between activities, behaviour and mood. Feasibility was asked about in terms of practicalities from both care receiver and provider prospective and in terms of policy. Participants were encouraged to give examples. Additionally, by looking at the context and experiences of depression we were able to examine whether BA would be likely to be acceptable and feasible in these settings. We have added more details about how acceptability and feasibility were explored in the interviews (lines 164-180). The results report on the findings from the interviews. In terms of feasibility space and resources were major concerns highlighted by participants, as explained in the discussion this echoes previous research (lines 746-749). We have added details making it more explicit how some of the practical constraints to BA can be overcome to improve feasibility (lines 749-751). We have also added to the discussion details on how BA is likely to be acceptable to participants as they did make explicit the link between behaviour and mood (697-700).

Reviewer 2

This paper reads extremely well. All sections are well written and the data collection and analysis were well executed. It would be helpful for the authors to consider dropping the aim and objectives - and rather merge it with the introduction. A table describing who was recruited and their demographics would have been helpful.

Thank you very much for your kind comments. As suggested we have merged the aim and objectives with the introduction (lines 90-109). We have also added a table describing people recruited and removed some of the details from the text (lines 148-157).

Line 408 is unclear - it would be helpful to recheck. It is not clear whether the psychologist and psychiatrist are trained or the providers are trained by the duo.

Thank you – this has been checked and clarified in the text (now line 438). It is the psychologists and psychiatrists that are trained.

Line 544 - it would be helpful to provide in addition an alternative name of Allah.

We have added "God" in brackets (now line 575)

It would have been helpful to explore the use of digital technology in delivering BA given the challenges with transportation etc.

We agree that this would have been important to explore and have added this as a limitation of the current work in the discussion (lines 765-766). We are already exploring this in related research by our group, we have added a reference to that work (line 767).
