# Supplementary material for: Living with depression and diabetes: A qualitative study in Bangladesh and Pakistan
Source: PLOS Glob Public Health. 2024 Jan 25;4(1):e0002846. doi: 10.1371/journal.pgph.0002846 (PMC10810509; doi:10.1371/journal.pgph.0002846)
Supplement: Attachment — Submitted filename: Response to Reviewers.pdf [file pgph.0002846.s003.pdf]

Responses to Reviewers

EDITOR:
I agree with the reviewer that a large part of the content does not have to do with the acceptability and feasibility of BA. I would suggest you revise the manuscript to add relevant content about acceptability/feasibility or reframe the focus of the paper so that it encompasses the actual content of the manuscript.

Thank you for your comments. In response to your suggestion and the comments made by reviewer 1 we have now reframed the paper to focus on the experiences of living with diabetes and depression, ways of managing diabetes and depression and treatment seeking behaviours rather than the acceptability and feasibility of BA. Views on BA are reported, and the paper discusses the integration of mental health in diabetes care more generally.

Specifically we have made the following changes to the paper:

- Title: the title has been changed from "Depression and Diabetes in Bangladesh and Pakistan: a qualitative study exploring the feasibility and acceptability of integrating Behavioural Activation for depression into diabetes care" to "Living with Depression and Diabetes: a qualitative study in Bangladesh and Pakistan"
- Abstract: the abstract has been shortened and reflects the reframing of the paper
- Introduction: it is explained that the study is part of a larger project that is adapting and testing BA in the context of Bangladesh and Pakistan, in this context this study is exploring experiences of living with diabetes and depression (lines 87-93)
- We have removed the original objectives 3 (to explore the feasibility and acceptability of BA) and 4 (to investigate opportunities to integrate BA into diabetes care). There is a new objective 3 which reads "to explore views on whether a brief therapy such as BA could be integrated into diabetes care in Bangladesh and Pakistan" (lines 100-101).
- Results: the third theme now reads "Views on BA" (rather than acceptability and feasibility of BA)
- Discussion: we have taken out sections about BA being feasible in this setting. We have added: participants appear open to mental health therapy and there is a need for mental health care (lines 721-722), practical steps to integrating mental health care more generally into diabetes care (lines 727-730) and that the DiaDeM feasibility trial is looking more in depth at the feasibility of BA (lines 746-747).
- Conclusion: has been shortened and rephrased to highlight the importance and challenges of integrating mental health care into physical health care more generally.

Reviewer 1:
Thanks to the authors for making some changes. Unfortunately, large parts of the qualitative analysis still cover experiences of living with diabetes and depression, coping styles and treatment seeking behaviours. The feasibility and acceptability aspects of BA are not the main ideas in the paper even now. In the responses, the authors have replied that they disagree with the fact that feasibility and acceptability have not been explored well. However, I still do not find these aspects covered adequately in the paper. What are the barriers and facilitators for

acceptable of BA methods. What are the experiences with implementing BA methods? These are not at all covered adequately even now. I dont think the paper convinces the reader that BA is feasible and acceptable to the community in Pakistan and Bangladesh.

Thank you for your comments. In response to your comments and the editor's we have reframed the paper to focus on the experiences of living with diabetes and depression, coping styles and treatment seeking behaviours rather than the acceptability and feasibility of BA. While views on BA are reported it is no longer suggested that it is the main focus of the paper.

At the time of data collection research participants had not had any experience of receiving or delivery of BA. We hope that our future publications, including results from the feasibility trial, will shed more light on the feasibility and acceptability of BA in these settings in Bangladesh and Pakistan.

The specific changes made are outlined above.

Reviewer 2:
No additional comments
